# *Clostridium difficile* Infections: A Global Overview of Drug Sensitivity and Resistance Mechanisms

**DOI:** 10.1155/2018/8414257

**Published:** 2018-02-21

**Authors:** Saeed S. Banawas

**Affiliations:** Department of Medical Laboratory Sciences, College of Applied Medical Sciences, Majmaah University, Majmaah 11952, Saudi Arabia

## Abstract

*Clostridium difficile (C. difficile)* is the most prevalent causative pathogen of healthcare-associated diarrhea. Notably, over the past 10 years, the number of* Clostridium difficile *outbreaks has increased with the rate of morbidity and mortality. The occurrence and spread of* C. difficile *strains that are resistant to multiple antimicrobial drugs complicate prevention as well as potential treatment options. Most* C. difficile *isolates are still susceptible to metronidazole and vancomycin. Incidences of* C. difficile* resistance to other antimicrobial drugs have also been reported. Most of the antibiotics correlated with* C. difficile* infection (CDI), such as ampicillin, amoxicillin, cephalosporins, clindamycin, and fluoroquinolones, continue to be associated with the highest risk for CDI. Still, the detailed mechanism of resistance to metronidazole or vancomycin is not clear. Alternation in the target sites of the antibiotics is the main mechanism of erythromycin, fluoroquinolone, and rifamycin resistance in* C. difficile. *In this review, different antimicrobial agents are discussed and* C. difficile* resistance patterns and their mechanism of survival are summarized.

## 1. Introduction

*Clostridium difficile* is a Gram-positive, anaerobic, spore-forming bacterium that is the most important causative pathogen of hospital-acquired diarrhea. Approximately 25% to 33% of antibiotic-associated diarrhea and 90% of pseudomembranous enteritis are caused by CDI [1]. According to the Centers for Diseases Control and Prevention (CDC), in the United States, CDI is responsible for more than 400,000 cases and 29,000 deaths each year and approximately more than $1 billion in additional medical budgetary costs [2, 3]. Thus, CDI is projected to become the most common healthcare-associated infection and hospital-acquired intestinal infection in the United States, Europe, and worldwide [4]. The clinical symptoms of CDI range from mild to severe diarrhea, which can lead to fulminant colitis, toxic megacolon, bowel perforation, sepsis, and ultimately death [5]. Although guidelines pertaining to the awareness, diagnosis, and treatment of CDI are available, the rate of CDI continues to increase in Europe and the United States [6].

The molecular study of the epidemiology of CDI has proved that a significant increasing number of outbreaks are caused by strains ribotype (RT) 027 and 078 [7]. In recent years, strain RT 027 has become the most prominent hypervirulent type that is responsible for severe infections and increasing mortality worldwide [8–13]. RT 078, another highly virulent strain, is found in European countries such as the Netherlands; causes infections in humans, particularly in the hospital and communities; and causes infections in animals [14–18].

CDI begins with an unusual exposure of the normal flora of the intestinal microbiota to antibiotics. This action results in a disruption of the normal intestinal microflora that allows for multiplication of* C. difficile* to cause disease [19]. Antibiotic resistance plays a crucial role in spreading CDI among hospitalized patients, particularly the elderly. Moreover, antibiotic resistance affects healthy people who have not been hospitalized or have not been submitted to antibiotic treatment and even pregnant women [20, 21].

In recent years, an increase in CDI has been reported in different countries. China, Sweden, and several other countries have reported a CDI incidence rate of ~17.1 cases/10000 admitted to a hospital [22]. CDI causes up to 30.7% of hospital infections and diarrhea cases. Additionally, RT 027 isolates are more common in the United States, Canada, and Poland [22–25]. The most important risk factor for CDI is broad-spectrum antimicrobial drugs that cause an imbalance of the intestinal microflora [26]. Several antimicrobial drugs such as erythromycin, penicillin, clindamycin, and fluoroquinolones cause CDI to spread more widely in and out of the hospital and increase the resistance of the epidemic strains of* C. difficile* to those drugs [27, 28]. Strain 027 is the most frequently observed drug-resistant RT strain and shows a statistically high incidence in Europe and the United States. Several countries have reported the resistance of strain 027 to moxifloxacin and, more recently, to metronidazole and vancomycin [23, 24, 28–35]. Consequently, this article discusses the general antibacterial drugs used to treat CDI, the CDI strains most resistant to drugs, and the strains' mechanism of resistance.

## 2. *C. difficile* Resistance to Antimicrobial Agents in Different Countries and Regions

The wide use of antibiotic in different countries with different treatments area causes a varying degree of drug resistance. In* C. difficile*, although all isolates are usually susceptible to metronidazole and vancomycin, resistance to other antimicrobials varies widely from one country to another (Table 1). Several studies have found that* C. difficile* is resistant to different antimicrobial agents in different countries (Table 1). Interestingly, the clinical isolates of* C. difficile* are still significantly sensitive to metronidazole and vancomycin, but there are reports of decreased susceptibility to metronidazole [24, 36–38]. Most of the antibiotics correlated with CDI, such as ampicillin, amoxicillin, cephalosporins, clindamycin, and fluoroquinolones, continue to be associated with the highest risk for CDI [27, 28]. The recommended treatment for primary and recurrent CDI involves the use of antibiotics with activities against* C. difficile,* including metronidazole, vancomycin, and fidaxomicin [27, 28]. Studies report that more than 30 hospitals around the United State have found the rate of resistance of* C. difficile* to clindamycin in around 36% (Table 1) [29]. However, the rate of resistance is higher in European and Asian countries. The resistance rates of* C. difficile* to clindamycin in Spain, Poland, and New Zealand are approximately 74%, 65%, and 61%, respectively (Table 1) [39–41], whereas the rates of resistance of* C. difficile* to clindamycin in China, Japan, Korea, and Iran are approximately 73.5%, 87.7%, 81%, and 89.3%, respectively (Table 1) [42–44].* C. difficile* has developed a high resistance to another antimicrobial drug, moxifloxacin. In the United States and Canada, the rates of resistance to moxifloxacin are approximately 36% and 83%, respectively (Table 1) [29, 45]. A recent study in Germany reported that the rate of resistance to moxifloxacin can be as high as 68% (Table 1) [30]. Spain's resistance rate to moxifloxacin is 43%, whereas Poland reported a resistance rate of nearly 100% (Table 1) [24, 40]. Recent studies have found that in Brazil and Israel the resistance rates against moxifloxacin are approximately 8% and 4.76%, respectively (Table 1) [46, 47].

The epidemic strains of* C. difficile* are more resistant to antibacterial drugs than are the nonepidemic strains. A study reported that, among 508 strains of* C. difficile*, PCR ribotype found that RT 027 was the most common, accounting for 28.1% of the isolates (143/508) [29]. The rates of resistance of* C. difficile* to clindamycin and moxifloxacin are ~50.3% and ~92.3%, respectively [29]. Approximately 39.1% of the 027 strains have a lower susceptibility to vancomycin (the Minimum Inhibitory Concentration (MIC_90_) 4 *μ*g/ml) [29]. Note that MIC results are based on Clinical and Laboratory Standard Institute (CLSI) document M11-A8 [29, 48, 49]. Another study confirms that the resistance rates of epidemic strain 027 to clindamycin, moxifloxacin, and erythromycin are higher than those of non-027 strains [33, 50]. In Asia, the RT 027 strain is rare, but the Korean epidemic strains RT 018 and 017 are resistant to clindamycin and moxifloxacin, significantly more so than nonepidemic strains [51, 52].

## 3. Resistance Mechanism of* C. difficile* to Most Commonly Used Drugs Recommended for CDI Treatment

### 3.1. Metronidazole

Metronidazole is a nitroaromatic drug that requires reduction of the 5-nitro group of its imidazole ring to become cytotoxic to bacterial cells [56]. Metronidazole is the first-choice antimicrobial drug for treating mild to moderate CDI [57]. Although the resistance rate of* C. difficile* strains to metronidazole is very low (Table 1), several studies have reported treatment failure after an antibiotic course of metronidazole [57, 58]. Moreover, the majority of clinical* C. difficile* isolates are still highly sensitive to metronidazole* in vitro* [59]. Recent studies have found that in Israel the rate of resistance of* C. difficile* against metronidazole is approximately 20.25% (Table 1) [46].

Furthermore, 12% of clinical isolates showed a heterogeneous resistance to metronidazole in 2008 [60] and were correlated with clinical failure. Approximately 25% of* C. difficile* epidemic 001 strains showed decreased sensitivity to metronidazole after a minimum inhibitory concentration (MIC 4 to 8 *μ*g/ml) was given [61]. In China, 18 isolates from Shanghai diarrhea patients with* C. difficile* were heterogeneously resistant to metronidazole [36]. In fact, most countries' criteria for* in vitro* drug susceptibility MIC results are based on the American Clinical and Laboratory Standards Association (CLSI) standards [48].* C. difficile* can be classified as resistance to metronidazole if the MIC ≥ 32 *μ*g/ml, but the breakpoint for reduced susceptibly is based on serum treatment concentration rather than intestine treatment concentration [48]. In contrast, the European Society for Clinical Microbiology and Infection (ESCMID) guideline for resistance of* C. difficile* to metronidazole is mainly based on activity in the intestine. Since 2012, the reduction point has been >2 *μ*g/ml for drug resistance [57, 62, 63]. Therefore, the European Antimicrobial Susceptibility Test Committee (EUCAST) standards for detecting metronidazole resistance in* C. difficile* are significantly higher than those reported in the current literature.

EUCAST and CLSI guidelines are the most popular breakpoint guidelines used in antimicrobial susceptibility testing worldwide. However, there are some significant differences between these two organizations that should be highlighted. Both methods have standardized (e.g., metronidazole) susceptibility test of anaerobic bacteria. According to their guidelines, anaerobic bacteria were classified as susceptible if the metronidazole MIC ≤ 8 *μ*g/ml; intermediate, if the MIC = 16 *μ*g/ml; or resistant if the MIC ≥ 32 *μ*g/ml, using agar dilution method (CLSI book guidelines, 2012). At least three different methods were used for* C. difficile* test: CLSI agar dilution method and the agar incorporation methods used by [64] and Etest. MIC values are higher with the agar incorporation methods compared with agar dilution methods, and the lowest MICs are achieved using Etest [65, 66]. Moreover, the susceptibility breakpoint recommended by CLSI and EDCAT differs. For example, the EUCAST breakpoints are susceptibility to metronidazole, MIC ≤ 2 *μ*g/ml, and resistance to metronidazole ~MIC ≥ 2 *μ*g/ml. These issues should be taken into consideration when comparing data from studies or country region utilized different susceptibility testing methods. Perhaps the major reason could be that all the breakpoints are based on serum drug concentrations but that effective antimicrobial therapy of CDI requires bactericidal intracolonic concentrations. Thus, clinical breakpoints are needed to be revised to help in predicting outcome and help to detect resistance mechanisms.

The bactericidal mechanism of metronidazole is not yet fully understood. Perhaps metronidazole metabolites are nonspecifically bound to bacterial DNA, inhibit bacterial DNA synthesis, and break the DNA strand to cause bacterial death. Furthermore, the metronidazole susceptibility and resistance mechanisms of* C. difficile* and other anaerobic bacteria are not yet clear. Researchers speculate that metronidazole may be reduced inside the cell. Increased metronidazole activation blocks bacteria's capacity for DNA repair [67].

### 3.2. Vancomycin

Vancomycin is the first antibiotic developed to treat moderate to severe CDI [57, 58]. The vancomycin structure contains a glycosylated hexapeptide chain and aromatic rings cross-linked by aryl ether bonds and exhibits poor absorption in the gastrointestinal tract [68]. To date, only in Poland have researchers reported three strains of* C. difficile* that are resistant to vancomycin, using the disc diffusion method [69]. Two additional methods have been used to detect vancomycin susceptibility: the agar dilution method and the Etest method. According to the European Committee on Antimicrobial Susceptibility Testing (EUCAST), reduced susceptibly to vancomycin is defined as an MIC of >2 *μ*g/ml. Recent studies have found that in Brazil and Israel approximately 58% and 31.5% of strains, respectively, are resistant to vancomycin (Table 1) [46, 47].

Vancomycin inhibits the biosynthesis of peptidoglycan by binding to D-alanyl-D-alanine at the end of the bacterial peptidoglycan precursor, which inhibits the synthesis of cell wall peptidoglycans [70].* Enterococcus *spp. and* Staphylococcus *spp. are the most common bacteria with resistance to vancomycin. The reason is the synthesis of D-alanyl-D-lactic acid, which replaces the normal cell wall peptidoglycan end of D-alanyl-D-alanine such that vancomycin is not able to bind the target (VanA, etc.). Another possibility is that synthetic D-alanyl-D-serine replaces the normal cell wall structure (VanE, etc.). Nevertheless, the mechanism of resistance in* C. difficile *is still unclear.

### 3.3. Fidaxomicin

In 2011, fidaxomicin was listed in the United States and Europe as a new treatment against CDI [52, 54]. Fidaxomicin acts through the inhibition of RNA polymerase during bacterial transcription. The sequence resulting from PCR amplification displayed a null mutation in the* rpoC *of the resistant strain [71]. Studies determined that the sequenced laboratory-induced-fidaxomicin-sensitive strains (MIC of 1 to 4 *μ*g/ml) contained Quot,* rpoB* (K1073H, Q1074K, Q1074H, V1143G, and/or V1143D) or* rpoC* (I10R, R89G, Q781R, and/or D1127E);* rpoB *and* rpoC* are the coding genes of RNA polymerase *β* subunit and *β*′ subunit, respectively [72]. Interestingly, the abovementioned results confirm that target site modifications in the RNA polymerase subunit of* C. difficile* against fidaxomicin are the suggested mode of resistance. In addition, the null mutation in the CD22120 gene that is homologous to the transcriptional regulator MarR family was found in a susceptible experimental strain [72], but the significance of the mutation remains to be further studied.

### 3.4. Erythromycin and Clindamycin

Erythromycin and clindamycin are members of the macrolide-lincosamide-streptogramin B (MLSB) family of protein synthesis inhibitors. Macrolide-lincomycin-type drugs are currently reported in countries exhibiting high resistance rates [73]. These drugs act on the bacterial ribosome 50S subunit and target the synthesis of bacterial proteins by inhibiting the extension of the peptide chain. A recent study found that clinical pathogens are resistant to erythromycin and clindamycin, mainly through ribosomal target changes or active efflux, while inactivated enzyme production can also cause bacteria to be resistant. The associated ribosomal methylase, encoded by the* ermB* gene, can modify ribosomal 23S rRNA, leading to high levels of resistance to these drugs [74]. The* ermB *gene is divided into different classes according to its sequence similarity, and its subtype now includes more than 20 species [75].

*C. difficile* resistance to erythromycin is encoded by the* ermB* gene located on the mobilizable conjugative transposon termed Tn5398 [75, 76], and researchers have reported that the* ermB* gene is located at other sites [77]. The* ermB* gene determines the polymorphism of a given region; for example,* ermB* in Europe determines the genetic structure of E4 and E15, which are the most common. Furthermore,* erm* gene-negative* C. difficile* can also show high levels of erythromycin resistance; however, the mechanism is unknown, particularly in which part of the strain 23S rDNA nucleotide substitution (C→T) at position 656 occurs [78].

### 3.5. Tetracycline

Recent studies have demonstrated that* C. difficile* resistance to tetracycline varies among different countries from 2.4% to 62.7% (Table 1) [32, 33, 38, 79–81]. Tetracycline is a rapid bacteriostatic agent that binds specifically to the axon initial segment of the bacterial ribosome 30S subunit, preventing aminoacyl-tRNA from binding to ribosomes, thereby inhibiting peptide chain elongation and protein synthesis. The mechanism of resistance to tetracycline by clinical pathogens is mainly associated with tetracycline resistance protein (tetM) and the active efflux system (tet A, B, C, D, etc.).

*C. difficile* is resistant to tetracycline, mostly by producing ribosomal protective protein (TetM), usually found on a conjugative* Tn916*-like element [81–83]. It was found that tetramine-resistant strains of tetracycline-resistant tetracycline were mostly transported by the Tn5397 transposon, whereas tetramine 017 and 078 were mostly located on* Tn916*-like transposons [84, 85].

### 3.6. Fluoroquinolones

Fluoroquinolones belong to a family of broad-spectrum antibiotics and are strongly linked to CDI [86, 87]. The widespread prevalence of strain 027 may be associated with resistance to fluoroquinolones. Many countries, such as New Zealand, Sweden, China Taiwan, France, and Germany, have* C. difficile* fluoroquinolone resistance rates between 7% and 40%; some resistance rates even exceed 80%, such as those of strains isolated from a multi-institutional outbreak in Quebec, Canada (Table 1) [88–91]. Fluoroquinolones act on either bacterial DNA gyrase and/or topoisomerase IV, leading to a cleavage of enzyme-DNA complexes and to inhibition of bacterial DNA synthesis [92].

The resistance mechanism of bacteria to fluoroquinolones is generally caused by two main mechanisms: (1) alteration of the drug target by a mutation in the encoding genes, leading to reduced affinity for the drug; and (2) either an increase in the active efflux of the drug or a decrease in permeability [92]. Previous studies have shown that* C. difficile*'s resistance to quinolone is due to changes in DNA gyrase subunit GyrA and/or GyrB [92]. These mutations can be found in the quinolone-resistance-determining region (QRDR) [92]. T82I is the most common site of mutation in GyrA. D71V, T82V, D81N, A83V, A118V, and A118T are the most common mutation sites in GyrB. In addition to these, D426N, D426V, R447K, R447L, S416A, E466K, A503S, S366A, and D501E are also the susceptible sites for mutations in GyrB [69, 93, 94].

### 3.7. Rifamycin Class

Two clinical members of rifamycin drugs are used for the treatment of CDI, rifamycin and rifaximin [95]. In 2009, a study reported that, in the United States and Canada, epidemic strains of* C. difficile* were resistant to RIF (approximately 7.9%) (Table 1) [23]. A study conducted in Spain reported that RIF resistance was ~24% (Table 1) [40]. Researchers from Hungary reported that the resistance rate of* C. difficile* was ~11.5%–14.9% from 2008 to 2010 (Table 1) [54, 55]. In 2010, a study in Poland reported a high rate of resistance of* C. difficile* to RIF of approximately 80% (Table 1) [53]. In China and South Korea, the resistance rate of* C. difficile* to RIF is quite low compared with that in Europe countries (~19.8%), and strains resistant to RIF (MIC ≥ 32 *μ*g/ml) have also been shown to be resistant to RFX [36, 51].

The major resistance mechanism of RIF is specifically associated with a mutation in DNA-dependent RNA polymerase beta subunit (RpoB), and RIF binds to form a stable complex that inhibits polymerase activity and inhibits DNA transcription.* C. difficile* and other Gram-positive bacteria resistant to RIF are also associated with a point mutation in RpoB. Recent studies have identified eight different RpoBs in* C. difficile*. All eight of the identified mutations involve substitutions between amino acids 488 and 548. R505K is the most common single substitution, usually leading to high levels of resistance (MIC ≥ 32 *μ*g/ml). By comparison, H502N shows a slightly lower MIC (MIC < 32 *μ*g/ml). Double substitution has also been described, such as R505K being combined with H502N or R505K being combined with I548M [96, 97].

## 4. Conclusion

The rate of* C. difficile* resistance to antimicrobials is rapidly growing worldwide. CDI has become a major concern for public health officials. Infections caused by* C. difficile *are unique because of their increased incidence as well as the increased use of certain antibiotics. Although some antibiotics are active and can be used to treat infections caused by* C. difficile, *the limitations of using these antibiotics have become a great concern due to increasing resistance in this pathogen. Further investigation is needed to understand the drug resistance mechanism of* C. difficile*, particularly to metronidazole, because it will provide new targets and new ideas for the development of antimicrobial agents and preventing CDI. Additionally, more attention is required at the clinical level regarding the strengthening of* C. difficile*'s resistance to avoid the blind use of broad-spectrum antimicrobial drugs, which should decrease the number of occurrences and the spread of drug-resistant* C. difficile* infections.

## Figures and Tables

**Table 1:** Resistance rates of *Clostridium difficile* to common clinical antimicrobial susceptibility agents in different countries.

Country (year)	Number of clinical isolates	Percentage of clinical isolates resistance rate to							Susceptibility methods	References
		Metronidazole	Vancomycin	Clindamycin	Erythromycin	Tetracycline	Moxifloxacin	Rifampicin		
Japan (2005)	73	0.0	0.0	87.7	87.7	0.0	0.0	0.0	Etest^1^	[43]
Germany (2009)	34	0.0	0.0	0.0	76	0.0	68	0.0	Etest	[30]
Sweden (2009)	80	0.0	0.0	65	13.8	7.5	15	3.8	Etest	[39]
N. America (2009)	316	0.0	0.0	41.5	0.0	0.0	38	7.9	Etest	[23]
France (2009)	224	0.0	0.0	34.8	19.2	0.0	8	0.0	Etest	[31]
Spain (2009)	154	0.0	0.0	74	49	0.0	43	24	Etest	[40]
Korea (2009)	120	0.0	0.0	81	80	0.0	42	0.0	AD^1^	[42]
China (2010)	110	0.0	0.0	88.1	85.3	62.7	61.8	29.1	Etest	[36]
S. Korea (2010)	131	0.0	0.0	67.9	0.0	0.0	62.6	19.1	Etest	[51, 53]
Poland (2010)	17	0.0	0.0	100	100	0.0	100	0.0	Etest	[53]
Hungary (2010)	288	0.0	0.0	29.5	31	0.0	41.2	26.4	Etest	[54, 55]
United States (2011–2013)	312	0.0	13.2	36	0.0	6.7	36	11.2	Etest	[29]
New Zealand (2011)	101	0.0	0.0	61	2	0.0	0.0	0.0	Etest	[41]
N. America (2012)	83	0.0	0.0	27.7	85.5	2.4	83	18	Etest	[33]
Poland (2012)	316	0.0	41.5	38	7.9	0.0	0.0	0.0	Etest	[23]
Iran (2013)	75	0.0	0.0	89.3	57.3	0.0	0.0	0.0	Etest	[44]
Hungary (2014)	200	0.0	29.5	31	21.5	11.5	0.0	0.0	Etest	[55]
Czech Republic (2015)	20	0.0	0.0	10	100	0.0	100	65	Etest	[11]
Brazil (2016)	50	0.0	58	0.0	0.0	0.0	8	0.0	AD	[47]
Israel (2017)	81	20.25	31.5	0.0	0.0	0.0	4.76	0.0	Etest	[46]

^1^Etest: Epsilometer test,  ^1^AD: agar dilution.

## Abbreviations

*C. difficile*: *Clostridium difficile*
CDI: *C. difficile* infection
RT: Ribotype.
